# Unfolding Role of a Danger Molecule Adenosine Signaling in Modulation of Microbial Infection and Host Cell Response

**DOI:** 10.3390/ijms19010199

**Published:** 2018-01-09

**Authors:** Jaden S. Lee, Özlem Yilmaz

**Affiliations:** 1 Department of Oral Health Sciences, College of Dental Medicine, Medical University of South Carolina, 29425 Charleston, SC 29425, USA; 2 Department of Microbiology and Immunology, Medical University of South Carolina, 29425 Charleston, SC 29425, USA

**Keywords:** purinergic signaling, danger signal adenosine, adenosine receptor, CD39/CD73, opportunistic infections, persistent pathogens, chronic inflammatory diseases

## Abstract

Ectonucleotidases CD39 and CD73, specific nucleotide metabolizing enzymes located on the surface of the host, can convert a pro-inflammatory environment driven by a danger molecule extracellular-ATP to an adenosine-mediated anti-inflammatory milieu. Accordingly, CD39/CD73 signaling has been strongly implicated in modulating the intensity, duration, and composition of purinergic danger signals delivered to host. Recent studies have eluted potential roles for CD39 and CD73 in selective triggering of a variety of host immune cells and molecules in the presence of pathogenic microorganisms or microbial virulence molecules. Growing evidence also suggests that CD39 and CD73 present complimentary, but likely differential, actions against pathogens to shape the course and severity of microbial infection as well as the associated immune response. Similarly, adenosine receptors A_2A_ and A_2B_ have been proposed to be major immunomodulators of adenosine signaling during chronic inflammatory conditions induced by opportunistic pathogens, such as oral colonizer *Porphyromonas gingivalis.* Therefore, we here review the recent studies that demonstrate how complex network of molecules in the extracellular adenosine signaling machinery and their interactions can reshape immune responses and may also be targeted by opportunistic pathogens to establish successful colonization in human mucosal tissues and modulate the host immune response.

## 1. Introduction

Purines are heterocyclic aromatic molecules that are among the most ancient and key compounds in the history of evolutionary biology [1]. Adenosine, one of the purine nucleosides composed of adenine and d-ribose, has been well recognized as molecular building block of the genetic code or as part of adenosine triphosphate (ATP)—the universal energy source of biological reactions [2]. Beyond these well-known roles, Alan Drury and Albert Szent-Györgyi from the University of Cambridge first proposed in 1929 that purines could also function as extracellular signaling molecules, which subsequently led to the discovery of extracellular receptors that mediate the signaling effects of purines [3,4]. In parallel, active research had been generating confirmatory results indicating the significance of extracellular adenosine and the mechanisms by which the molecule takes effect. It has also become well established that adenosine is not only a metabolite of ATP, but also an endogenous purine nucleoside and a danger molecule that can alert the host immune system [5]. 

Lately, various physiopathological stimuli that elicit molecular signaling events in control of cellular injury have been found to be regulated by adenosine signaling including sustained low-level of inflammasome activation, cell death, and organelle functions [6,7,8,9]. Adenosine as a host derived small molecule appears to assume a significant role in reshaping the microenvironment during inflammatory processes as well as microbial assaults specifically through the interaction with widely expressed four subtypes of cell surface G-protein-coupled adenosine receptors named A_1_, A_2A_, A_2B_, and A_3_ [10,11,12]. Extracellular adenosine has also been shown to take part in anti-inflammatory immune reaction that is most characterized by reduced proliferative responses of immune cells as well as attenuated innate and adaptive immunity [13,14]. Some recent studies also propose that adenosine signaling may yield varying inflammatory responses depending on the amount of time post-injury [15,16,17]. During cell stress or infection, extracellular nucleotide and nucleoside levels, such as adenosine or the danger molecule extracellular ATP (eATP), are regulated by a variety of specific enzymes to ensure proper purinergic signaling [14]. The interrelation between extracellular adenosine and eATP is based on the presence of two subtypes of purinergic receptors, P1 for adenosine and P2 for eATP, that are mostly co-expressed by both immune and non-immune cells [17,18]. The majority of the characterized roles of eATP are pro-inflammatory in nature and involve activating the host immune system via P2X_7_ receptor, such as the induction of interleukin-1β and interleukin-18 (IL-1β and IL-18), modulation of host cell death, immune cell proliferation, and clearance of pathogenic microorganisms [14]. Rapid release of eATP immediately after an injury has been shown to have excitatory effects on immune cells, in particular, rampant pro-inflammatory cytokine releases. In contrast, reduced eATP signaling in conjunction with increased activation of adenosine receptors A_2A_ and A_2B_ has been shown to aid in limiting duration and intensity of inflammation [17]. 

Given these recent findings, there has been a steady movement towards examining the importance of extracellular adenosine signaling for understanding the balance between the “immune-activation” and “immune-suppression” [19,20]. For both eATP and adenosine as host-derived small danger molecules, insignificant amounts are detected in healthy conditions without having substantial immunologic functions. However, when present at a higher concentration, these molecules are strongly associated with sequential triggering of a variety of immune molecules and cells [10,21]. Therefore, maintaining observed equilibrium appears to critically contribute to fine-tuning the host response against harmful cellular/molecular events, such as microbial assaults, by limiting prolonged or uncontrollable inflammatory damage by pathogen to the host cells [22]. However, the specific molecular events that orchestrate the extracellular adenosine synthesizing pathway and their ability to preferentially modulate on host selective defense mechanisms, especially, towards the pathogenic microorganisms are poorly understood. There is still a relative lack of knowledge about specific functions of the members of the purinome that are suggested to participate in the adenosine signaling axis especially in the context of host–microbe interplay. These include eATP-to-adenosine converting host surface ectonucleotidases CD39 and CD73 as well as their interaction with the adenosine cell membrane receptors. 

Few groups have pointed out previously the potential immunologic function of adenosine signaling during host–microbial interactions [23]. However, the research endeavors have usually revolved around the signaling pathway in specialized immune cells [24]. In this review, we first provide a fundamental overview on the generation of extracellular adenosine and its key regulators, CD39, CD73, and other partnering molecular pathways. The role of extracellular adenosine as an emerging immunomodulatory mediator in host–pathogen interaction and the subsequent modulation of both host and microbial responses will be the center of our comprehensive discussion. We will also examine the immunologic function of the nucleotide metabolism specifically relating to adenosine at the epi-mucosal tissues because of its budding role in the host protective mechanism against infections by opportunistic pathogens. Lastly, we will discuss the potential of clinical translation output for adenosine signaling molecules as future therapeutic targets to manage inflammatory conditions associated with opportunistic pathogens and dysregulated immune responses. 

## 2. The Basics of Extracellular Adenosine Metabolism and Signaling

**Figure 1:**
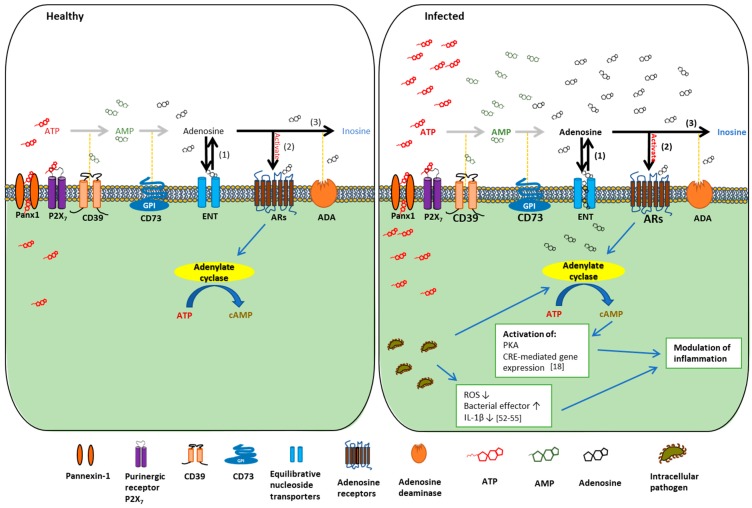
The change in the metabolism of adenosine signaling between the states of healthy and infected. Adenosine signaling is a crucial contributor to the innate host defense mechanism. Extracellular ATP (eATP) is released to the extracellular space through Pannexin-1 channels (Panx-1). Once in the extracellular space, eATP may activate the P2X_7_ purinergic receptor or may be converted to adenosine by the CD39/CD73-mediated adenosine pathway. Extracellular adenosine can be metabolized into three separate ways: (1) be transported back into the cell via bi-directional equilibrative nucleoside transporters (ENT) in the direction of concentration gradient; (2) activate adenosine receptors (ARs) on the plasma membrane with the stimulation of P2X_7_; or (3) be degraded into inosine via adenosine deaminase (ADA). During infection, a substantial increase in the amount of extracellular ATP released by host cells is observed across many species of microbes [23,50,51]. Subsequently, the level of extracellular adenosine and activation of ARs may be altered, potentially changing the outcome of their intracellular signaling cascades. Mucosal pathogens such as *P. gingivalis* can promote activation of anti-inflammatory ARs. This will further result in elevated cAMP formation via adenylate cyclase activity and subsequent activation of protein kinase A (PKA) and cAMP response element (CRE)-mediated gene expression that are shown to non-redundantly down-regulate inflammation [18]. *P. gingivalis* has also been shown to attenuate host oxygen-reactive-species (ROS) production and IL-1β release, while increasing bacterial effector secretion [52,53,54,55]. These specific molecular events described above culminate in the modulation of inflammation, which may lead to change the course and severity of infection.

Adenosine signaling, including extracellular purine nucleotide metabolism, extracellular adenosine generation, and subsequent adenosine receptor activation, has been shown to be as complex as many other metabolic pathways. Here, we aim to provide a detailed synopsis of the adenosine signaling by discussing a series of key modulators involved in this highly regulated pathway. There is evidence that extracellular adenosine can originate from the intracellular space through multiple paths [25,26]. Intracellular ATP can be released to become extracellular ATP—a “danger” molecule released from damaged/stressed host cells or upon infection by pathogens [27]—that undergoes ATP hydrolysis mainly by the host surface enzymes, CD39 (ectonucleoside triphosphate diphosphohydrolase-1; ENTPD1) and CD73 (ecto-5′-nucleotidase; NT5E). Alternatively, intracellular adenosine may also be transported into the extracellular space via two families of bidirectional transporters: concentration-based transporters and equilibrium-dependent transporters [28]. However, many studies have indicated that the predominant source of adenosine in the extracellular milieu originates from the breakdown of eATP during disease conditions, such as inflammation and ischemia [29,30,31]. Metabolic disturbances or other types of cellular damaging events to the host are associated with leakage or regulated release of ATP from intracellular to extracellular compartment [32,33]. eATP along with adenosine diphosphate (ADP) is subsequently converted to adenosine monophosphate (AMP) by CD39, which is ultimately metabolized to adenosine by CD73 [30,34,35]. Extracellular adenosine has been shown to play a significant role in several physiological/pathological processes by activating adenosine receptors such as A_1_, A_2A_, A_2B_, and A_3_ [13,36,37,38] (Figure 1). Because individual adenosine receptors can result in different biologic functions, it is important to note that the consequences of extracellular adenosine signaling may heavily depend on the relative expression pattern of the adenosine receptors on the extracellular surface of specific cell types or tissues [17,39]. In pathophysiological conditions such as inflammation, transcriptional changes in the expression of adenosine receptors have been reported to alter the downstream host danger signaling events through extracellular adenosine [40,41]. Furthermore, the extracellular adenosine concentration at the cell surface is another major determinant of molecular signaling effects as individual adenosine receptors have varying affinities to adenosine [42]. Once activated, downstream signaling occurs through the changes in adenylate cyclase activity, leading to subsequent alteration of intracellular cyclic AMP (cAMP) levels as a second messenger [43] (Figure 1).

When extracellular adenosine completes its task as a danger signal molecule, the signaling can be terminated by the uptake of adenosine from the extracellular to the intracellular compartment [44]. This termination process is another highly orchestrated biological process with multiple steps regulated independently on a transcriptional level [45,46,47]. The adenosine uptake from the extracellular to intracellular space is mainly through equilibrative nucleoside transporter 1 (ENT1) and equilibrative nucleoside transporter 2 (ENT2), diffusion-limited channels that allow adenosine to freely cross the cellular membrane according to its concentration gradient [47] (Figure 1). ENTs are widely expressed in vascular endothelia, epithelia, erythrocytes, or immune cells [39]. Under physiologic conditions, differences between intra- or extracellular adenosine concentrations are insignificant. Therefore, the net flow through the ENTs is minimal. In contrast, when extracellular adenosine concentrations are substantially elevated during pathogenic states, adenosine flux through the transporters is specifically directed from the extracellular space toward the intracellular compartment [46] (Figure 1). The function of ENTs in adenosine signaling has been supported by several studies. For example, genetic deletion of ENT1 in mice was shown to result in significantly higher hepatic adenosine levels after liver ischemia when compared to wildtype mice [48]. The ENT1-deficient mice also showed markedly attenuated liver inflammation induced by ischemia by measuring of interferon-γ (IFN-γ) and IL-6 protein levels and neutrophil marker myeloperoxidase (MPO) in livers [48]. Pharmacologic inhibition of ENTs with dipyridamole has also been demonstrated to induce elevated accumulation of extracellular adenosine and prevent reactive oxygen species generation by neutrophils [49]. These studies collectively support the direct interaction of ENTs with the danger molecule adenosine, which may alter the extent of inflammation.

## 3. The Roles of the Main Regulators of Extracellular Adenosine Signaling in Regulating Microbial Infection and Inflammation

### 3.1. CD39 and CD73

It is well recognized that the ATP hydrolysis pathway by CD39 and CD73 results in the conversion of a pro-inflammatory stimulus (eATP) into an anti-inflammatory mediator (adenosine). These cell membrane ectonucleotidases are often referred to as immunological switches [19]. The expression and activity of CD39 and CD73 have been reported to change dynamically in various pathophysiological contexts [56], including infection by microbial organisms [57,58,59]. For years, CD39 and CD73 have been mainly studied in cancer research, and a relatively consistent pattern of change in the expression or activity of the ectonucleotidases was observed. On the contrary, only a cadre of researchers have reported their studies on adenosine produced by CD39 or CD73 expression in the context of host immune response, specifically caused by microbial agents [60,61,62]. During infection, extracellular adenosine signaling may be construed as damaging to the host as it suppresses the pro-inflammatory response, which is considered important to combat the invading microbial pathogens during the subacute stage of infection [17]. However, the complexity of the outcome of these host responses has been shown to largely depend on the type and stages of infection. Here, we distinctively describe studies that provide novel insights into the molecular mechanisms of action and contribution of each ATP-to-adenosine-converting ectonucleotidase to modulation of infection severity and associated host immune responses. 

A study by Théâtre et al. sought to examine whether extended elevation of CD39-mediated nucleotide metabolism in mouse airways is sufficient to cause an inflammatory response to bacterial challenge by *Pseudomonas aeruginosa* [61], one of the major mucosal opportunistic pathogens causing chronic lung infection that ultimately leads to morbidity and mortality of patients with cystic fibrosis [63,64]. Firstly, no development of spontaneous lung inflammation was confirmed in transgenic mice overexpressing human CD39 via lung histology and inflammatory cell counts. Thereafter, both wildtype and transgenic mice were challenged with bacterial lipopolysaccharide (LPS) through intratracheal installation. Based on the subsequent histological examination of lung sections from both groups, more pronounced leukocyte infiltration, alveolar edema, and congestion were observed in transgenic mice lungs. In addition to the pro-inflammatory phenotypes for LPS-treated transgenic mice, LPS administration significantly increased the production level of inflammatory chemokines/cytokines such as keratinocyte chemoattractant (KC), regulated on activation normal T-cell expressed and secreted (RANTES; CCL5), and IL-6. When live *P. aeruginosa* was introduced to the same set of transgenic mice to reproduce the pro-inflammatory phenotype of the LPS-treated transgenic mice, higher numbers of inflammatory cells were recruited into the lung. Similarly, with LPS, whole live bacteria-induced secretion of KC and RANTES was increased in transgenic mice. Furthermore, transgenic mice were found to show enhanced elimination of *P. aeruginosa* than wildtype mice by counting live microorganisms in the lungs. These findings suggest that stimulated expression of CD39 by LPS or microbial instillation to animals in lung epithelia may augment innate host defenses and promote immune cell recruitment for effective pathogen elimination [61]. The exact mechanism for these outcomes still needs to be determined; however, this study highlights the putative pro-inflammatory role of CD39 and suggests that future investigations need to consider tissue specificity, expression levels, amount and/or duration of adenosine generation, and specific microorganisms that encounter the host before the role of CD39-mediated adenosine signaling especially in host–pathogen interaction can be fully elucidated. 

CD39 has also been implicated in the context of adaptive immunity. Regulatory T cells (Tregs) balance normal tissue homeostasis by mediating antigen-specific immune responses [65]. During chronic infections, the induction of Tregs appears to limit inflammatory responses, thereby reducing host tissue damage [66]. However, pathogens may also be not fully eradicated, allowing further establishment of persistent infection [65]. Sufficient evidence is present suggesting that *Mycobacterium tuberculosis* simultaneously modulates the innate and adaptive immune response during its microbial establishment in host [67]. A study using *M. tuberculosis* infected mice showed that the infection triggered antigen-specific CD4^+^ Tregs, which delayed adequate pathogen clearance and favored persistent infection [68]. CD39 expression has also been confirmed in CD4^+^ Tregs [69]; however, further investigation is required to unveil the potential role of CD39 in CD8^+^ Tregs during *M. tuberculosis* infection. Compared to CD4^+^ Tregs, CD8^+^ Tregs have been far less extensively studied and are known for their classic function for long-term memory to previously encountered antigens along with their maintained effector functions [65]. Boer et al. described for the first time the functional expression of CD39 in human CD8^+^ T cells activated by *M. tuberculosis* [60]. The activation of CD8^+^ Tregs by treatment of live human *Bacillus* Calmette-Guérin (BCG) vaccine induced the expression of CD39 on the Tregs, which significantly co-expressed several well-known Treg markers CD25, Foxp3, LAG-3, and CCL4. Moreover, by using purified CD8^+^CD39^+^ Tregs, CD39 expression was found to be more than simply a marker for CD8^+^ Tregs. CD8^+^CD39^+^ Tregs showed a suppressive activity of CD4^+^ human T helper cell responder clone in contrast to CD8^+^CD39^−^ Tregs. This study elucidated that the involvement of CD39 in CD8^+^CD39^+^ Tregs is perhaps a key factor in regulating the extent of the host immune reactions against mycobacteria [60]. Taken together, the presence of CD39^+^ Tregs would suggest a specific function of importance for adenosine signaling involving in immune regulation against opportunistic chronic pathogens, such as *M. tuberculosis* in mucosal tissues. Furthermore, these findings suggest host adenosine enzymatic pathway molecules, such as CD39, can play direct roles during the onset of infection, where they also exert a strong influence on the generation of the adaptive immune responses.

Unfortunately, the investigation on the role of CD39 in the context of microbial infection is still in its infancy as the studies discussed above represent the majority of current literature on the topic. By introducing these studies, we hope to bring the attention to the potential of CD39 as an immunoregulator during microbial colonization and persistence. On the other hand, ectonucleotidases have been in the center of immuno-oncology research for decades [70,71,72]. Increased CD39 expression has been repeatedly reported in distinct types of tumors and cancer cell lines [71,72,73,74]. Moreover, CD39 as a generator of adenosine has been mainly considered anti-inflammatory in cancer research. For example, inhibiting the enzymatic activity of CD39 has been shown to alleviate the immunosuppressive function of tumors and cancer cell lines [74]. Based on the previous literature discussed above, it is worth noting that, while CD39 overexpression in infection led to the elevation of the innate immune response, comparable results were observed by blockade of CD39 in tumor cells. Therefore, it is tempting to speculate that CD39 may assume diverse roles depending on the disease pathology and microenvironment. Future studies may also determine whether this putative specificity is a consequence of direct cross-talk the CD39 receptor may engage in with different signaling partners on the host cell membranes, some of which may not have been characterized. 

Recently, the role of CD73 in host-derived immune response has been studied in the context of acute opportunistic microorganisms such as food-borne pathogen *Salmonella* spp., which is the leading cause of hospitalization and death among susceptible human population [75]. To prevent hospitalization and potential death, *Salmonella* infection must be controlled with a proper host response by well-coordinated action of innate and adaptive immunity [57]. The clearance of *Salmonella* requires IFN-γ production by CD4^+^ T cells and IL-17 from T helper 17 cells [76,77]. The outcome of infection is also dictated by the balance between CD4^+^ effector T cells and Tregs [78]. Recently, Alam et al. investigated the role of the ectonucleotidase CD73 in regulating inflammation and bacterial burden using CD73-deficient mice during *Salmonella* infection [57]. It was reported that CD73 expression in lymphocytes was significantly downregulated by *Salmonella* infection [57]. To understand the contribution of CD73 to the *Salmonella* virulence in host, murine splenocytes from uninfected or *Salmonella*-infected wild-type or CD73-deficient mice were isolated to yield cell lysates, some of which were treated with alpha, beta-methylene adenosine-5′-diphosphate (APCP), a CD73-specific inhibitor. Upon the treatment, the level of pro-inflammatory cytokines such as IL-17A and IFN-γ were elevated in the splenocytes and CD4^+^ cells. Addition of 5′-AMP, the substrate for CD73, in the culture suppressed the pro-inflammatory cytokines, which was partially restored by the addition of APCP. These results from the functional assays showed evidence that CD73-expressing splenocytes produce adenosine, which lowers the level of pro-inflammatory cytokines. In the same *Salmonella* pathogenesis study with in vivo CD73-deficient mouse model, CD73-deficient mice had significantly higher expression of pro-inflammatory cytokines and reduced anti-inflammatory responses. The transgenic animal appeared to be more resistant to infection with greater inflammatory output and a significantly lower bacterial load in the liver compared to wild-type mice with intact CD73. Taken together, the results suggest that human pathogen, *Salmonella*, interferes with the innate CD73 signaling that seems to serve to initiate special adaptive immune responses. This specific cross-talk between the innate and adaptive systems in turn may help the pathogen to increase its microbial colonization and prolong the infection course in host [57]. As CD73 is one of the rate-limiting factors in extracellular adenosine generation pathway, the ability of Tregs to generate adenosine from ATP and ADP appears crucial to the suppression of effector cells during infection.

Alam et al. also studied the expression of CD73 on human T helper (Th) cells, and its role in regulating *Helicobacter felis*-induced gastritis and the bacterial colonization [62]. In this study, it was first established that CD73 is functionally expressed in gastric Th cells, which was followed by assaying cell cultures for IFN-γ secretion with or without using APCP. The results showed that the addition of APCP in a coculture assay with Tregs and effector Th cells did not reverse the suppressive action of Tregs on proliferation. In another experiment, anti-CD3/CD28–stimulated autologous peripheral blood mononuclear cells were cocultured with Tregs with or without 5′-AMP, a substrate of CD73. The addition of APCP in this assay did reverse the suppressive activity of Tregs. These data from the functional studies showed that the gastric Th cells may be able to generate adenosine to downregulate IFN-γ production. In the same study, an in vivo mouse model was also utilized to investigate the role of CD73 in the development of gastritis and bacterial colonization. When CD73-deficient mice were infected with *Helicobacter felis*, a well-known model of opportunistic *H. pylori* infection in humans, more severe forms of gastritis were observed in the mice with alongside elevated mRNA levels of IL-2, Tumor necrosis factor-α (TNF-α), IFN-γ and impaired Treg function in gastric tissue compared to wild-type mice [62]. The study concluded that CD73 expressed in gastric Th cells contributes to local adenosine accumulation and the control of inflammation associated with infection. Furthermore, reduced production of adenosine in CD73-deficient mice showed an association with impaired Treg function, increased gastric inflammation, and reduced levels of bacterial colonization [62]. Thus, the results point to the growing notion that CD73 plays a critical role by generating adenosine to limit inflammation and modulate the level of *Helicobacter* infection. 

Another study recently reported that reduced production of adenosine by both pharmacologic inhibition of CD73 and the use of CD73-deficient mice dramatically increased murine resistance to *Streptococcus pneumoniae,* an opportunistic mucosal pathogen of human [79]. First, wild-type C57BL/6 mice were injected with APCP followed by *S*. *pneumoniae* challenge. At Day 3 after infection, mice injected with APCP showed significantly increased bacterial burden in the lungs than the mock-treated mice. Similarly, when S. *pneumoniae* was introduced to wild-type and CD73-deficient mice, the level of bacterial counts was significantly higher in the mice with CD73 deficiency compared to the wild-type mice. These results collectively point out the significance of CD73 in S. pneumoniae lung infection. Furthermore, polymorphonuclear leukocytes (PMNs) isolated from the blood and bone marrow of mice treated with APCP displayed a significant defect in killing of pneumococci as compared to PMNs from mock-treated C57BL/6 mice. Comparable results were found using mice treated with APCP. These findings suggest that extracellular adenosine alters the bactericidal function of PMNs, one of the major initial line defense cells of the innate immune system, to clear the *S. pneumoniae* infection [79]. The mechanism by which extracellular adenosine produced by CD73 mediates endothelial transmigration of PMNs during pneumococcal infection could potentially involve chemotactic signals and/or molecules that directly modulates PMN-endothelial cell interactions. With presence of extracellular adenosine, production of both chemokine IL-8 by endothelial monolayers and C-X-C motif ligand 2/3 (CXCL2/3) (murine paralog of IL-8) was diminished. On activated PMNs, adenosine seemed to inhibit up-regulation of the β2 integrin (CD11b/CD18), which majorly participates in neutrophil adhesion to endothelium, transendothelial migration, and phagocytosis [36]. It was also found that following the pneumococcal infection, the level of pulmonary IL-2 was significantly elevated in CD73-deficient mice compared to wild-type mice. These results suggest the critical role of extracellular adenosine in modulating the function and/or recruitment of PMNs which may modify the outcome of *S. pneumoniae* infection. 

Since CD73 is more immediate to the production of extracellular adenosine, far more studies have been conducted on the role of CD73 within host–pathogen interaction compared to CD39. Interestingly, opportunistic infection by both well adapted microbes such as *H. pylori* and *Salmonella* spp. displayed a significantly reduced bacterial colonization in CD73-deficient mice [57,62]. These findings suggest that CD73 can decrease host inflammatory responses that are designed to combat against pathogenic organisms and thereby inadvertently promote those microorganisms to establish infection in host tissues. Although it has not yet been confirmed with further studies, it is tempting to speculate that CD73-mediated adenosine signaling is specifically targeted by host-adapted pathogens. Therefore, CD73 may play a key role in bacterial growth and colonization of host tissues, such as epi-mucosal tissues, by opportunistic pathogens in the commensal flora [57,62,79]. Moreover, given the roles characterized in studies described above, CD39/CD73-mediated adenosine signaling pathway may function as a connector between the innate and adaptive immunity and may aid opportunistic microorganisms to evade multi-level host defenses for successful persistence. Thus, CD39/CD73 adenosinergic machinery may be targeted to develop therapeutics to limit/eliminate overt or chronic infection caused by opportunistic pathogens.

### 3.2. Adenosine Receptors

There have been numerous studies contributing to the characterization of G-protein coupled surface adenosine receptors [13,79,80,81,82,83]. Adenosine receptor A_1_ (A_1_AR) is the most abundant and largely expressed with particularly high presence in inflammatory cells such as neutrophils [37,84]. A_1_AR is known to modulate the activity of adenylate cyclase, the enzyme responsible for increasing cAMP. Adenosine receptor A_2_ (A_2_AR) is more widely distributed than A_1_AR and can be further subdivided into A_2A_ (A_2A_AR) and A_2B_ (A_2B_AR) based on high and low affinity for adenosine, respectively [85]. Ohta and Sitkovsky discovered for the first time that A_2A_AR has a non-redundant role in the attenuation of inflammation and tissue damage using A_2A_AR-deficient mice, suggesting that A_2A_AR is a critical part of the physiological negative feedback mechanism for limitation and termination of both tissue-specific and systemic inflammatory responses [86]. In other studies, activation of adenosine receptorA_3_ (A_3_AR) has been found to inhibit adenylate cyclase and directly stimulate phospholipases C and D in mammalian brain, representing an early response to ischemia [87,88]. In comparison with the other adenosine receptors, A_3_AR shows drastic species differences in structure, tissue distribution, and its functional and pharmacological properties between human and rat [87]. A_1_AR and A_2A_AR are described to have a high affinity for adenosine, while A_2B_AR and A_3_AR show significantly lower affinity for adenosine. Activation of A_1_AR occurs at 0.3–3 nM concentration of extracellular adenosine, A_2A_AR at 1–20 nM, while A_2B_AR or A_3_AR activation requires an agonist concentration larger than 1 μM [89]. There is growing evidence suggesting adenosine receptors are fully expressed in immune cells including neutrophils, macrophages, dendritic cells and mast cells [90]. In addition to the immune cells, several studies have documented the functional expression of adenosine receptors in the human intestinal and gingival epithelial cells as well as human gingival fibroblasts [11,91,92]. Human primary gingival epithelial cells express functional adenosine receptors A_1_, A_2A_, A_2B_, and A_3_ [11]. A_2A_AR inhibition studies showed that A_2A_AR may be utilized to induce an anti-inflammatory effect by *Porphyromonas gingivalis*, a major opportunistic periodontal bacterium, for its prolonged persistence in the oral mucosa [11]. In the study of mice intestine, A_2A_AR and A_2B_AR were reported to be the predominant adenosine receptors limiting the immune pathology and mucosal dysbiosis during acute toxoplasmosis; this suggests that anti-inflammatory adenosine receptor activation may constitute an effective approach to control inflammation in the gut associated with reduced availability of adenosine [58,91]. The expression of adenosine receptors A_1_, A_2A_, and A_2B_, but not A_3_, was also confirmed in human gingival fibroblasts (HGFs) via RT-PCR analysis [92]. Agonists for A_1_AR and A_2A_AR were shown to synergistically increase IL-1β-induced IL-6 and IL-8 production in HGFs, which suggests possible involvement of adenosine signaling in the regulation of inflammatory responses in periodontal tissues [92]. Along with this study, a few reports have recently introduced the idea that specific AR signaling can trigger pro-inflammatory actions particularly in a chronic aspect of inflammatory diseases, which will need further investigation. Given the growing evidence that depicts the importance of ARs in regulation of inflammation, the notion of targeting adenosine signaling therapeutically for the treatment of several chronic and systemic diseases, such as asthma, arthritis, and cancer has been increasingly supported [93,94,95,96].

Although there have been studies investigating the functional importance of adenosine receptors in various conditions [13,79,80,81,82,83,88], the specific effectors or the detailed mechanism(s) by which adenosine and AR signaling contribute to the innate immune response in the context of pathogen survival and persistence has yet to be firmly established. Previous studies investigating the putative mechanism(s) have placed their emphasis on either of these two factors: immunosuppressive effects of extracellular adenosine molecules during infection or specific adenosine receptor activation to differentially mediate the downstream host responses. Specific human disease states associated with defects or mutations of any of the four adenosine receptors are actively under investigation; however, the majority of adenosine receptors’ exact biologic and cell-specific functions have been identified under diseased conditions [87,97,98,99]. Of the four adenosine receptors, the potential roles of A_2A_AR and A_2B_AR have been most extensively studied in contributing to an anti-inflammatory environment. Similarly, these ARs are recently explored as immune modulators that directly promote the host microbial colonization and growth by infectious organisms in a wide range of disease states [11,58,100,101]. 

For example, Crane et al. studied enteropathogenic *Escherichia coli* (EPEC), a common cause of diarrhea in children in developing countries, and reported that, when adenosine was exogenously introduced to various human epithelial cell lines, the EPEC triggered a large release of ATP from the host cells and subsequent increase in the breakdown of the eATP to adenosine by CD39 and CD73 mediated hydrolysis [102]. It was also shown that adenosine produced from the breakdown of eATP triggered a vigorous chloride secretory response in intestinal tissues, which were studied in rabbit distal colon (in the Ussing chamber model) and T84 cell monolayers [102]. The most significant aspect of the study was to demonstrate that even without the presence of immune cell recruitment, large concentrations of adenine nucleotides (ATP, ADP, AMP, adenosine) can be released directly from EPEC-infected intestinal host cells and trigger fluid secretion, which presents several crucial features. Specimens from human EPEC infection cases mostly show highly dispersed EPEC infection with several normal areas [103]. Furthermore, EPEC is shown to prefer villi for its adherence, whereas crypt cells are the ones responsible for secretory action to generate a watery diarrhea [104]. Therefore, it may be unlikely that the intestinal cells adhered to by EPEC have the capacity to generate fluid secretion as previously thought. This report suggests a new paradigm proposing that adenine nucleotides released from EPEC-infected cells act paracrine way on uninfected cells nearby, including crypt cells, for generating a secretory response in the intestine [102]. A follow-up study was performed examining the direct effect of adenosine on EPEC. It was reported that adenosine stimulated EPEC growth in several types of media in vitro, led to a more diffuse adherence pattern of the pathogen, and changed the pattern of expression of several virulence genes including *espA* and *espB* [105]. In vivo, when adenosine levels were reduced by addition of exogenous adenosine deaminase (ADA) in the rabbit intestine, the number of EPEC bacteria recovered was decreased by over 10-fold. Conversely, inhibitors of ADA increased EPEC-induced fluid secretion and the number of EPEC bacteria recovered from intestinal fluid while promoting the in vivo expression of EPEC secreted proteins [105]. Taken together, it was suggested that extracellular adenosine may serve as an immunomodulator that regulates pathogenic bacterial growth and metabolism through its specific signaling events.

*P. gingivalis*, a Gram-negative opportunistic pathogen of the human oral cavity, has been strongly associated with severe forms of periodontal disease and recently named a major risk factor in severe systemic chronic conditions such as rheumatoid arthritis, Type 2-diabetes, and orodigestive cancers [106,107,108]. *P. gingivalis* has been shown to utilize multiple mechanisms to successfully colonize, replicate, and disseminate within and through human mucosa (primary gingival epithelial cells, GECs), which is the initial target of the microorganism in the oral cavity [54,55,109]. Interestingly, studies of *P. gingivalis* have discovered that *P. gingivalis* can secrete an effector molecule Nucleoside-Diphosphate-Kinase (Ndk), and this effector enzyme interferes with P2X_7_ receptor- eATP coupling and contribute to extracellular adenosine formation through eATP hydrolysis [52,110]. This ability of *P. gingivalis* to scavenge danger signal eATP has been shown to greatly serve the microorganism for host survival and persistence [50,53,55] (Figure 1). It was also recently shown that GECs express a family of all members of adenosine receptors and that treatment of *P. gingivalis*-infected GECs with A_2A_AR-specific agonist CGS-21680 resulted in markedly elevated intracellular bacterial replication in these epithelial cells [11]. Furthermore, A_2A_AR-specific antagonist and knockdown via RNA interference significantly reduced metabolically active intracellular *P. gingivalis*. Following A_2A_AR selective agonist stimulation, significantly higher levels of an anti-inflammatory mediator cAMP were induced during *P. gingivalis* infection [11]. These recent data collectively suggest that inhibition of inflammation through A_2_AR may result in a microenvironment conducive to bacterial growth. It has also been suggested in this study that the adenosine signaling pathway through A_2A_AR might be subverted by *P. gingivalis* for its successful host cell survival in the oral mucosa [11]. 

*Chlamydia trachomatis* species are one of the most common bacterial causes of sexually transmitted disease (STD) in humans [111]. Much of the pathology seen during *Chlamydia* infection is attributed to the inflammatory response from the host. Like other opportunistic bacterial pathogens known to establish chronic infections in mucosal tissues, *Chlamydia* infection has also been associated with adenosine receptor activation. It was reported that persistent infection of cervical epithelial cells by *C. trachomatis* was dependent on A_2B_AR activation [112]. In addition, A_2B_AR was discovered to be the predominant adenosine receptor responsible for *Clostridium difficile*-induced inflammation in the intestinal epithelium [101]. When human gut epithelial cells were challenged with *C. difficile* toxins, upregulation of A_2B_AR was observed. Moreover, the blockade or deletion of A_2B_AR decreased *C. difficile*-induced tissue injury and improved the outcome of the infection, suggesting the pathogen-favorable function of A_2B_AR [101]. Another study investigating A_2B_AR further showed an increased clearance of *Klebsiella pneumoniae* in A_2B_AR-deficient mice, which suggests that the presence of A_2B_AR signaling specifically could be exploited by opportunistic microorganisms as a mechanism to evade host pathogen killing and promote Gram-negative bacterial pneumonia in host mucosal tissues [113]. Endotoxin-induced lung injury studies in murine models also demonstrated a significant role of A_2B_AR in down regulating lung inflammation in LPS-induced lung injury via a combination of pharmacological inhibition and genetic elimination approaches [114]. Human microvascular endothelial cells used for this study showed increased A_2B_AR expression at both gene and protein level when exposed to inflammatory stimuli including Prostaglandin E2, IL-1β, IL-4, and IL-6 over a time-course of up to 24 h. Similarly, the induction of A_2B_AR expression was also observed via immunohistochemistry of lungs from mice exposed to LPS and showed robust increases in A_2B_AR staining on pulmonary epithelial or endothelial structures [114]. Collectively, these findings suggest that during host–pathogen interactions, A_2B_AR activation appears to work in favor of pathogen survival due to its anti-inflammatory effect. However, it is important to note that adenosine receptors, including A_2B_AR, have been found to be increasingly expressed following the exposure to external inflammatory stimuli because of their non-redundant roles in protecting tissue from further damage.

The recently published studies introduced in this review collectively support the notion that extracellular adenosine often assumes a role as an immune regulator molecule to selectively modulate the host immune system and reshape the course and/or severity of chronic infections by opportunistic pathogens. Even though various species of microorganisms were used in these studies, the addition of adenosine into their experimental model consistently led to enhanced replication and extended survival of the microorganisms in their host environment. The elevated level of adenosine has been repeatedly found in different types of pathogenic states including inflammation, hypoxia, and trauma [115]. Interestingly, some bacteria including *Staphylococcus aureus* and *Bacillus anthracis* have been documented to have an adenosine synthase homologue, a cell membrane-anchored enzyme that metabolizes AMP to adenosine [116]. The metabolite then functions as a virulence factor by introducing an additional source of anti-inflammatory adenosine, which aids the pathogen with circumventing phagocytic clearance attempts by the host during cellular infection [116]. Based on these findings, it is tempting to suggest that opportunistic microorganisms have devised diverse pathogenic molecular mechanisms to promote the generation of extracellular adenosine in the host and to generate additional adenosine via their own adenosine synthase mechanisms, when equipped, as part of their microbial existence and host colonization strategies. 

The roles of adenosine signaling in the immune system for controlling of inflammatory response and the targeting of specific molecules in the extracellular adenosine signaling cascade by microorganisms to evade of host pathogen elimination have recently gained increasing attention. The potential use of adenosine receptor-based therapies in the treatment of chronic inflammatory conditions associated with opportunistic microbes has presented an exciting opportunity to identify how these therapies may be applied. However, to facilitate the drug development, many more specific studies on host–pathogen interaction need to be conducted to better understand the exact role of extracellular adenosine and to dissect out any variability in the affinity of receptors across various species.

## 4. Diagnostic and Therapeutic Potential of Adenosine Receptor Signaling in Treating Chronic Inflammatory and Infectious Diseases

The interest in therapeutic potential of adenosinergic compounds including the receptor agonists and antagonists has been developing for over a decade [42,94,117]. For a long time, the development of synthetic adenosine agonists for clinical applications had remained trivial. However, consistent approach to create selective agonists and antagonists facilitated research on therapeutic approaches of modulating the adenosine receptors and their functions. To name a few current established applications, the activation of A_1_AR has been implicated in the widely used treatment of supraventricular tachycardia via Adenocard^®^. A_2A_AR-activating Adenoscan^®^ is known to induce vasodilation for cardiac imaging [118]. A_2B_AR and A_3_AR selective agonists and antagonists are also being investigated in clinical trials for asthma and autoimmune inflammatory disorders, respectively [119,120].

Adenosine receptor agonists and antagonists with high potency and selectivity have been synthesized for all four subtypes, with selective A_2B_AR agonists being the most recently reported [118]. Some of these ligands are specific for an individual subtype, whereas others have mixed specificity for multiple subtypes. With the great advances in the development of pharmacological tools for investigating the adenosine receptors, the lack of selective ligands is not the biggest challenge, but rather it is the broad distribution of the receptors and/or observed variation in affinity for a specific compound at the same subtype in different species of organisms [121,122]. Another important factor to consider is the varying expression levels of adenosine receptor subtypes in tissues [11]. Strict cautions must be taken in generalizing the potential of a given compound based on certain in vitro and/or in vivo animal models for clinical applications. 

Of the four adenosine receptors, A_2A_AR has been identified as the most promising therapeutic target for controlling inflammation [123]. While there is compelling evidence for further investigation on the anti-inflammatory role of A_2A_AR in modulating infectivity and persistence of pathogenic microorganisms, numerous reports support the receptor’s role as a regulator of inflammatory response [42,100,124]. Previous studies have shown that inhibition of inflammation is achieved by treatment with selective A_2A_AR agonists that are known to have anti-inflammatory and immunosuppressive effects [125]. Several A_2A_AR-selective agonists including UK-432097, sonedenoson, and binodenoson have been clinically evaluated [118]. However, even with many studies suggesting high potential of adenosine receptors as therapeutic targets, only one agent, regadenoson (Lexiscan^®^) has been able to reach the clinical usage so far. This may be due to the challenge with systemic applications of the A_2A_AR-selective agonists lacking receptor selectivity [126]. Therefore, efforts have been directed to creating A_2A_AR agonists with site-specific actions and local delivery systems. For example, 5′-phosphate prodrugs of A_2A_AR agonists are under development, which are to be preferably cleaved to release the A_2A_AR-specific agonist at sites of inflammation where CD73 is highly expressed [127]. Given the current challenges, more refined strategies need to be formulated to produce an adenosine receptor agonist with a superior therapeutic index for yielding significant clinical benefits without evoking further undesired immune reactions.

## 5. Concluding Remarks

Extracellular adenosine is one of the significant biomolecules that can accumulate in the inflammatory environment with pleiotropic effects on the host. In recent years, the identification of an increasing number of human pathogens’ interaction with host cell surface ectonucleotidases CD39 and CD73 that convert eATP into adenosine has raised intriguing questions regarding the role of these enzymes for microbial colonization as well as their infectious pathology. Moreover, the subsequent extracellular adenosine signaling via its receptors has been potentially suggested to be a non-redundant underlying mechanism behind the pathogen evading host immune response/killing and establishing successful pathogen survival in host. While further work is in need to elucidate exact functions of multiple mediators of the adenosine signaling events, the studies outlined in this review support the notion that extracellular adenosine generated by CD39 and CD73 and its subsequent activation of certain subtypes of adenosine receptors may lead to a serious modulation of inflammatory responses, which can favor bacterial life and broaden the impact of infection (Table 1). In addition, the focus of the previous studies unveiling the connection between adenosine signaling and infection has been primarily on the innate immunity components. In contrast, this review illuminates the idea that CD39/CD73/adenosine receptor-mediated signaling events may play a critical role in orchestrating a specific adaptive immune response. 

As mentioned earlier, due to the widely spread distribution of adenosine receptors, their agonists may have universal effects in multiple tissues producing a variety of responses. It is challenging to implement these agonists with high specificity, particularly in microbial diseases and/or chronic inflammatory conditions associated with microbial etiology. Moreover, activation or blockade of adenosine receptors may yield varying effects in different cells within the same tissue and in different stages of a disease process. Therefore, it appears vital to have a further understanding of both the targeted disease process and the complexity of adenosine signaling in different cellular environments and conditions. 

## Figures and Tables

**Table 1:** Overview of examples of different cell types reported to: (1) express CD39, CD73, and/or adenosine receptors; and (2) have association with microbial infection. Based on the up-to-date literature, notable changes in inflammatory responses as well as innate and/or adaptive immune responses that occur by altered adenosine signaling during infection are also summarized. Nicotinamide adenine dinucleotide phosphate-oxidase (NADPH oxidase); Cyclic AMP (cAMP).

Cell locations	Cell Types	CD39 Expression	CD73 Expression	Expressed Adenosine Receptor	Relevant Microbe	Adenosine Signaling in Infection		References
						Inflammatory Response	Other Immune Response	
Epithelial tissue	Airway/Bronchi	Expressed	Expressed	A_1_ A_2A_ A_2B_ A_3_	*Pseudomonas aeruginosa* *Streptococcus pneumoniae*	Pro-inflammatory cytokine levels	Infiltration of m macrophages and neutrophils Pathogen killing of PMNs	[61,79,128,129,130]
	Gingiva	Expressed *	Expressed *	A_1_ A_2A_ A_2B_ A_3_	*Porphyromonas gingivalis*	Anti-inflammatory cytokine levels Inflammasome activation	Modulation of NADPH oxidase signaling and cAMP generation	[11,53,54,55,107,108,109,110,111,131]
	Colon (T84)	Expressed	Expressed	A_2A_ A_2B_	*Helicobacter pylori*	Pro-inflammatory cytokine levels	cAMP generation	[57,91,124]
	Cervix (HeLa 229)	Highly expressed	Not known	A_2B_	*Chlamydia trachomatis*	Not known	cAMP generation	[112]
Immune tissue	Regulatory T cells	Highly expressed	Highly expressed	A_2A_ A_2B_	*Mycobacterium tuberculosis*	Not known	CD4^+^ T helper-1 cell responses	[60,67]
	Splenocytes	Expressed	Expressed	A_2A_	*Salmonella* spp.	Pro-inflammatory and anti-inflammatory cytokine levels	Pathogen clearance ability of host	[57,75,132]

* = not published.
